# Application of the modified Framingham cardiovascular risk score to newly diagnosed type 2 black African diabetic patients

**Published:** 2007

**Authors:** Andre Pascal Kengne, Mesmin Dehayem, Simeon Pierre Choukem, Paschal Awah, Jean-Claude Mbanya

**Affiliations:** George Institute for International Health, University of Sydney, Australia; Department of Internal Medicine and Specialities, Faculty of Medicine and Biomedical Science, University of Yaounde 1; Yaounde Central Hospital, Internal Medicine Unit: Service of Endocrinology, Yaounde, Cameroon; Department of Internal Medicine and Specialities, Faculty of Medicine and Biomedical Science, University of Yaounde 1; Yaounde Central Hospital, Internal Medicine Unit: Service of Endocrinology, Yaounde, Cameroon; Department of Internal Medicine and Specialities, Faculty of Medicine and Biomedical Science, University of Yaounde 1; Yaounde Central Hospital, Internal Medicine Unit: Service of Endocrinology, Yaounde, Cameroon; Department of Internal Medicine and Specialities, Faculty of Medicine and Biomedical Science, University of Yaounde 1; Yaounde Central Hospital, Internal Medicine Unit: Service of Endocrinology, Yaounde, Cameroon

## Abstract

**Background and objective:**

Cardiovascular complications are a major cause of morbidity and mortality in people with type 2 diabetes. The aim of this cross-sectional study was to assess the baseline cardiovascular risk of newly diagnosed type 2 diabetic patients, using the modified Framingham point-score scale.

**Methods:**

Data on cardiovascular risk factors were collected from 97 consecutive newly diagnosed type 2 diabetic patients at the Yaounde Central Hospital, Cameroon. Projected 10-year cardiovascular risk was estimated for each patient using the modified point score of Framingham.

**Results:**

Men and women were equally represented and the age of the participants ranged from 33 to 86 years. Mean values for total, low-density lipoprotein (LDL) and non-high-density lipoprotein (HDL) cholesterol were relatively elevated in the study population while HDL cholesterol levels were low. Total cardiovascular risk scores and the individual scores for each of the parameters in our model were significantly higher in females than in males. The 10-year risk for coronary artery disease was above 20% in 7.2% (6.7−7.7) of subjects, and between 10 and 20% in 21.7% (20.8−22.6). Overall, men were more at risk than women (*p* < 0.001).

**Conclusions:**

Patients with type 2 diabetes already had increased cardiovascular risk at clinical diagnosis in Cameroon. There is therefore considerable need for cardiovascular risk-factor intervention, particularly for hypertension and obesity, as well as dyslipidaemia, along with tight metabolic control.

## Summary

Cardiovascular diseases account for up to 80% of the excess risk of death in people with type 2 diabetes.^1^ Diabetics share the same cardiovascular risk factors as non-diabetics, however, these risk factors seem to be more deleterious in people with diabetes. Evidence from large-scale clinical trials has confirmed the beneficial role of adequate blood-pressure and lipid-lowering treatments, and anti-platelet therapy on the reduction of cardiovascular risk in patients with diabetes.^2^ For those apparently free of vascular complications, risk tables are regularly used to predict the risk of the future development of cardiovascular events, and to decide on the possibility of cardio-preventive treatment.^3^

Cardiovascular risk assessment engines are based on population studies, and some of these have included people with diabetes. Risk engines specific to diabetics also exist.^4^-^6^ Risk assessment builds on classical cardiovascular risk factors to predict the subsequent development of cardiovascular events in a given individual. The ultimate goal of these equations is to select eligible patients in routine practice for more aggressive cardiovascular risk intervention, thereby increasing the effectiveness of primary prevention. They also help to increase a patient’s adherence to prescribed prevention measures.

Despite their effectiveness, most of these tables tend to underestimate the cardiovascular risk of people with diabetes.^4^,^7^ Many alternatives have been proposed to minimise the discrepancy. These include the development of risk-assessment tools based exclusively on diabetics, accounting for other parameters such as triglycerides, left ventricular hypertrophy, and novel or emerging cardiovascular risk factors.^8^ Systematic statin and anti-platelet treatment for everyone with type 2 diabetes without consideration of individual risk estimation is increasingly being promoted, based on the results of preventive intervention studies. 9 Cost constraints limit the implementation of such recommendations in resource-poor settings where access to routine care is already a major challenge.

More realistic approaches aimed at identifying the diabetics most likely to benefit from preventive cardiovascular interventions should therefore be encouraged in this context. Such approaches are further supported by the recent reports indicating that cardiovascular risk is increased mostly in diabetics with multiple cardiovascular risk factors.^10^ Moreover, cardiovascular risk is significantly influenced by the duration of diabetes, being lower at the early stage of the disease, and increasing as it progresses.

We undertook this cross-sectional study to assess the initial cardiovascular risk profile of newly diagnosed type 2 diabetic patients in Cameroon, using the modified Framingham pointscore table.

## Patients

This cross-sectional study was conducted at the outpatient department of the Endocrine and Diabetes Service of the Yaounde Central Hospital, a tertiary institution of the capital city of Cameroon with a catchment population of close to three million, and representing all social and ethnic classes in the country. A total of 97 consecutive newly diagnosed type 2 diabetic patients were received during the 16-month study period from October 2002 to February 2004. Patients with acute type 1 presentation including diabetic ketoacidosis and heavy ketonuria were excluded. All patients were considered for the evidence of diabetes based on their medical records, interviews and onsite capillary blood glucose determinations. Known duration of less than three months was the criterion for newly diagnosed cases. The 1999 WHO diagnostic criteria were used throughout the study.

Ethical approval was obtained from the ethics committee of the Faculty of Medicine and Biomedical Sciences prior to investigation. Reported diagnosis of a lipid disorder or nephropathy was recorded. Other past medical history included self-reported diagnosis of hypertension and the known duration, smoking and alcohol consumption.

## Methods

Anthropometric measurements including height, weight, waist and hip circumferences were assessed using standardised methods. Blood pressure (BP) was measured on the right arm after the patient had rested for five minutes, using automated blood pressure machines (OMRON® M4). The mean of two readings taken two minutes apart was used. Pulse pressure was obtained by subtracting the diastolic from the systolic blood pressure. Body mass index (BMI) was calculated as weight (kg) divided by the square of the height (m^2^) and patients were classified for BMI status as normal (BMI < 25 kg/m^2^), overweight (25 ≥ BMI < 30 kg/m^2^) and obese (BMI ≥ 30 kg/m^2^). Waist-to-hip ratio was defined as waist circumference divided by the hip circumference.

Capillary blood glucose was measured using the OneTouch® (Lifescan, Johnson & Johnson, USA) blood glucose monitor. Biological investigations including lipid profiles (total cholesterol, HDL cholesterol and triglyceride levels), and serum urea and creatinine levels were obtained from the result sheets provided by the reference laboratory of Centre Pasteur du Cameroun as part of the routine initial investigations. LDL cholesterol was calculated using the Friedewald’s equation^11^ and the non-HDL cholesterol was obtained as the difference between total cholesterol and HDL cholesterol.

The total individual cardiovascular risk score was the sum of the scores obtained for each of the included parameters. The corresponding 10-year cardiovascular risk was obtained from the Framingham table.^12^

Data analysis was performed using the SPSS® software. Qualitative data were expressed as percentages and 95% confidence intervals where relevant; and quantitative variables as means and standard errors of the mean (SEM). The normal distribution of quantitative data was checked with the Kolmogorov-Smirnov test. The Student’s *t*-test and chi-square test (or likelihood ratio where relevant) were used appropriately to compare variables. A *p*-value less than 0.05 was considered significant.

## Results

In total, 97 newly diagnosed type 2 diabetic patients were received during the study period with men and women almost equally represented (women 52.6%). General characteristics of the study population are displayed in Table 1. A positive family history of hypertension and diabetes was found in 22.7 and 35.1%, respectively. No patient reported a previous diagnosis of lipid disorders or kidney disease. Age ranged from 33 to 86 years with an average of 55.98 years. The 21 (21.6%) patients currently smoking were predominantly men (71.4%, *p* = 0.012). Forty-eight (49.5%) patients consumed alcohol and were more likely to be male subjects (*p* = 0.015).

**Table 1:** Gender Distribution Of Clinical And Biological Parameters

*Variable*	*Men (n = 46)*	*Women (n = 51)*	*Total (n = 97)*	*p*
Age (years)	55.17 ± 1.76	56.71 ± 1.41	55.98 ± 1.11	0.49
Duration of hypertension (yrs)	1.72 ± 1.07	1.93 ± 0.65	1.85 ± 0.56	0.86
Body mass index (kg/m^2^)	26.54 ± 0.66	26.81 ± 0.98	26.68 ± 0.60	0.82
Waist circumference (cm)	94.02 ± 1.53	89.77 ± 1.87	91.76 ± 1.24	0.09
Hip circumference (cm)	100.93 ± 1.42	101.90 ± 2.02	101.44 ± 1.26	0.70
Waist-to-hip ratio	0.93 ± 0.009	0.88 ± 0.008	0.91 ± 0.007	0.002
Systolic BP (mmHg)	137.57 ± 4.03	141.58 ± 3.77	139.70 ± 2.75	0.47
Diastolic BP (mmHg)	86.48 ± 2.07	86.70 ± 2.08	86.60 ± 1.46	0.94
Pulse pressure (mmHg)	51.09 ± 2.81	54.88 ± 2.64	53.11 ± 1.92	0.33
Initial FCG (mg/dl)	323.27 ± 23.16	292.26 ± 20.84	309.10 ± 15.76	0.33
Confirmatory FCG (mg/dl)	236.83 ± 17.54	280.50 ± 36.56	255.94 ± 18.85	0.26
Total cholesterol (g/l)	1.79 ± 0.08	1.88 ± 0.05	1.83 ± 0.05	0.37
Triglyceride (g/l)	1.25 ± 0.15	1.10 ± 0.08	1.17 ± 0.08	0.40
LDL cholesterol (g/l)	1.13 ± 0.07	1.22 ± 0.05	1.18 ± 0.04	0.30
HDL cholesterol (g/l)	0.41 ± 0.03	0.44 ± 0.03	0.43 ± 0.02	0.57
Non-HDL cholesterol (g/l)	1.37 ± 0.08	1.44 ± 0.05	1.41 ± 0.05	0.49
Creatinine (mg/l)	11.83 ± 0.66	9.68 ± 0.56	10.85 ± 0.46	0.02
Urea (g/l)	0.30 ± 0.02	0.26 ± 0.02	0.28 ± 0.01	0.26

FCG: fasting capillary glucose; BP: blood pressure.

Unlike weight (*p* = 0.02), height (*p* < 0.001), waist-to-hip ratio (*p* = 0.002) and serum creatinine levels (*p* = 0.02), the distribution of clinical and biological parameters showed no significant difference between men and women. Women displayed relatively high figures for age, total cholesterol, HDL cholesterol and non-HDL cholesterol. Overall, mean values for BMI (26.68 kg/m^2^), and systolic (139.70 mmHg) and diastolic (86.60 mmHg) blood pressure were high compared to standard normal values.

The point scores for individual variables included in the model were high in women except for smoking and total cholesterol. The difference, however, reached significance only for systolic blood pressure (*p* < 0.001) and total cholesterol (*p* = 0.002) (Table 2). When alcohol consumption was taken into consideration, the points for total cholesterol (*p* = 0.008) and smoking (*p* = 0.021) were higher in alcohol consumers. In the same subgroup, the points for systolic blood pressure (*p* = 0.001), age (*p* < 0.001) and total cholesterol (*p* = 0.01) were significantly lower. BMI status had no significant influence on the distribution of points.

**Table 2:** Distribution Of Variable Dependent Points Score By Gender

*Variable (points for)*	*Men (n = 46)*	*Women (n = 51)*	*Total (n = 97)*	*p*
Age	6.15 ± 0.78	7.63 ± 0.62	6.93 ± 0.50	0.14
Systolic blood pressure	0.98 ± 0.14	2.78 ± 0.28	1.93 ± 0.18	< 0.001
Total cholesterol	1.63 ± 0.31	2.47 ± 0.30	2.07 ± 0.22	0.053
Smoking	1.04 ± 0.29	0.22 ± 0.09	0.61 ± 0.15	0.006
HDL cholesterol	1.15 ± 0.16	0.71 ± 0.18	0.92 ± 0.12	0.066
Points total	10.96 ± 0.70	13.80 ± 0.61	12.45 ± 0.48	0.003

The 10-year cardiovascular risk was generated independently of the diabetes status and is displayed in Table 3. Up to 7.2% [95% confidence interval (CI) 6.7−7.7] of patients had a 10-year risk greater than 20%. Another 21.7% (20.8−22.6) had a risk between 10 and 20%. The risk distributions between genders differed significantly (*p* < 0.001), and in particular, men were more at risk than women.

**Table 3:** Gender Distribution Of The 10-Year Risk Of Coronary Events

*Cardiovascular risk (%)*	*Men*		*Women*		*Total*	
	*n*	*% (95% CI)*	*n*	*% (95% CI)*	*n*	*% (95% CI)*
> 20	5	10.9% (9.9−11.8)	2	3.9% (3.4−4.4)	7	7.2% (6.7−7.7)
10–20	17	36.9% (35.1−38.6)	4	7.8% (7.0−8.6)	21	21.7% (20.8−22.6)
< 10	24	52.2% (50.1−54.3)	45	88.2% (85.6−90.8)	69	71.1% (69.4−72.8)

CI: confidence intervals.

## Discussion

We found in this study that up to 30% of newly diagnosed diabetics in our setting were at high risk of subsequent cardiovascular complications, based on the assessment model used. In addition, men had significantly higher risk than women. Cardiovascular complications are a major cause of death and diseases in people with type 2 diabetes. Compared to non-diabetics, people with diabetes are at increased risk of cardiovascular diseases and their prognosis is worse after a cardiovascular event.^13^

The increased susceptibility of diabetics to cardiovascular diseases can be explained at least in part by classical cardiovascular risk factors and other diabetes-related factors. This multifactorial nature must be accounted for when designing approaches targeting cardiovascular risk reduction in people with diabetes. However there is still lack of agreement as to the best strategies to target cardiovascular burden in diabetics.

Both the American Diabetes Association (ADA) and the National Cholesterol Education Program (NCEP) consider diabetes as coronary risk equivalent, hence their recommendation of treating everyone with type 2 diabetes as patients with a past history of cardiovascular events.^14^,^15^ Such strategies cannot easily be envisaged in developed countries where access to appropriate healthcare is already a major challenge, and where infectious diseases still prevalent are competing with emerging non-communicable diseases for the limited health resources. Even in affluent countries, issues relating to doctor−patient concordance will limit the implementation of such strategies, adding to the threat of risk compensation.

Despite the growing burden of diabetes in Africa, cardiovascular diseases, for many reasons, are still reported at a lower rate in this part of the world.^16^ Such reasons include the lack of risk-assessment tools adapted to the context of clinical practice in Africa where paraclinical investigations are not always available. The Framingham point score used in this study has the advantage of including few paraclinical parameters and can therefore be easily adapted to the context of Africa.

Age, total cholesterol and systolic blood pressure contributed most of the cardiovascular risks in this study. These are major cardiovascular risk factors both in diabetics and the general population, as is extensively described in the literature. Moreover, atherosclerotic vascular diseases tend to occur at a young age in diabetics. The high cardiovascular risk profile of men, as found in this study, has previously been reported. Apart, from possible gender-specific factors, smoking could also be a contributor. Smoking is a modifiable risk factor, and significantly alters the risk profile of diabetics. It must therefore be strongly addressed in this population, along with other traditional risk factors.

In a risk approach model that appropriately controls smoking, blood pressure and lipid disorders in our population, the 10-year risk will significantly drop in both men and women. In a resource-poor setting, this will constitute a substantial gain, especially when the available health facilities are not geared toward coping with attendant cardiovascular complications.

## Limitations

This study had some limitations that must be accounted for when considering our findings. The original Framingham point-score risk table was not developed for diabetics. Diabetes status in the development process of this tool was considered as a ‘coronary risk equivalent’, implying the management of diabetics as patients with a past history of coronary artery disease without further need for global risk evaluation. Such an approach, however, cannot be applied in the African context because of financial and logistical constraints, and most importantly, the lack of accurate data on the burden of cardiovascular diseases in diabetics in this part of the world.^16^ For similar reasons, diabetes-specific cardiovascular risk engines such as the UKPDS tool could not be applied in our study.^5^,^6^

Another limitation had to do with the fact that risk-score tools are specific to the population for which the tools were developed, and cannot easily be applied to different populations without recalibration. This recalibration requires cross-sectional data on cardiovascular risk factors and cardiovascular diseases that are not readily available in this context.

Using a control group would also have enabled us to ascertain whether risk status described here was specific to diabetes status. However, in a cardiovascular risk assessment in the general population in South Africa, Steyn *et al.*^17^ reported a 10-year risk lower than that reported in our study. If our populations are comparable, this will indicate that at diagnosis, people with diabetes have high cardiovascular risk.

## Conclusions

Cardiovascular diseases, although still reported to be of low prevalence, are becoming more widespread among diabetics in Africa. In this study, independent of the diabetes status, newly diagnosed diabetic patients and particularly men in Cameroon displayed a cardiovascular risk profile that warranted aggressive treatment. Age, high blood pressure and other classical risk factors were major contributors. There is, therefore, considerable need for cardiovascular risk intervention, particularly for blood pressure lowering, obesity as well as lipid disorders, along with a tight metabolic control at the early stage of diabetes in this population.

To overcome the limitations of this study and to produce more comprehensive and accurate estimates, cohort studies are urgently needed to monitor cardiovascular outcomes in diabetics and in the general population in sub-Saharan Africa. Representative cross-sectional assessment of risk factors would also enable development of risk-assessment tools specific to the population in this part of the world.
